# Maternal Valacyclovir and Infant Cytomegalovirus Acquisition: A Randomized Controlled Trial among HIV-Infected Women

**DOI:** 10.1371/journal.pone.0087855

**Published:** 2014-02-04

**Authors:** Alison C. Roxby, Claire Atkinson, Kristjana Ásbjörnsdóttir, Carey Farquhar, James N. Kiarie, Alison L. Drake, Anna Wald, Michael Boeckh, Barbra Richardson, Vincent Emery, Grace John-Stewart, Jennifer A. Slyker

**Affiliations:** 1 Department of Medicine, University of Washington, Seattle, Washington, United States of America; 2 Department of Epidemiology, University of Washington, Seattle, Washington, United States of America; 3 Department of Global Health, University of Washington, Seattle, Washington, United States of America; 4 Department of Biostatistics, University of Washington, Seattle, Washington, United States of America; 5 Department of Laboratory Medicine, University of Washington, Seattle, Washington, United States of America; 6 Department of Pediatrics, University of Washington, Seattle, Washington, United States of America; 7 Centre for Virology, Department of Infection, School of Biomedical and Life Sciences, University College London, London, United Kingdom; 8 Department of Microbial and Cellular Science, University of Surrey, Guildford, United Kingdom; 9 Department of Obstetrics and Gynaecology, University of Nairobi, Nairobi, Kenya; 10 Division of Vaccine and Infectious Disease, Fred Hutchinson Cancer Research Center, Seattle, Washington, United States of America; 11 Division of Clinical Research, Fred Hutchinson Cancer Research Center, Seattle, Washington, United States of America; Glaxo Smith Kline, Denmark

## Abstract

**Background:**

Studies in HIV-1-infected infants and HIV-1-exposed, uninfected infants link early cytomegalovirus (CMV) acquisition with growth delay and cognitive impairment. We investigated maternal valacyclovir to delay infant acquisition of CMV.

**Methods:**

Pregnant women with HIV-1, HSV-2 and CD4 count >250 cells/µl were randomized at 34 weeks gestation to 500 mg twice-daily valacyclovir or placebo for 12 months. Maternal CMV DNA was measured in plasma at 34 weeks gestation, in cervical secretions at 34 and 38 weeks gestation, and in breast milk at 7 postpartum timepoints; infant CMV DNA was measured in dried blood spots at 8 timepoints including birth.

**Results:**

Among 148 women, 141 infants were compared in intent-to-treat analyses. Maternal and infant characteristics were similar between study arms. Infant CMV acquisition did not differ between study arms, with 46/70 infants (66%) in placebo arm and 47/71 infants (66%) in the valacyclovir arm acquiring CMV; median time to CMV detection did not differ. CMV DNA was detected in 92% of 542 breast milk specimens with no difference in CMV level between study arms. Change in cervical shedding of CMV DNA between baseline and 38 weeks was 0.40-log greater in the placebo arm than the valacyclovir arm (p = 0.05).

**Conclusions:**

In this cohort of HIV-1-seropositive mothers, two-thirds of infants acquired CMV by one year. Maternal valacyclovir had no effect on timing of infant CMV acquisition or breast milk CMV viral loads, although it modestly reduced cervical CMV shedding. Maternal prophylaxis to reduce infant CMV acquisition warrants further evaluation in trials with antiviral agents.

**Trials Registration:**

ClinicalTrials.gov NCT00530777

## Introduction

In the setting of maternal HIV-1, infant CMV infection is associated with impaired growth and development [1] and HIV-1/CMV co-infected infants have a high risk of mortality [2], neurologic deficits [3], and HIV-1 disease progression [4]. Infants may acquire CMV *in utero*, during delivery, or postnatally through breast milk or saliva [5]. Maternal CMV antibodies protect against congenital disease and infection; but postnatal protection wanes rapidly [6]. In sub-Saharan Africa, >80% of children acquire CMV during the first year of life [5], and acquisition may occur earlier if mothers have HIV-1 [2].

We evaluated maternal prophylaxis with valacyclovir as a potential intervention to delay infant CMV acquisition. Valacyclovir is often used to suppress genital herpes simplex type-2 (HSV-2) infection during pregnancy, and acyclovir has been used successfully at high doses to reduce CMV reactivation in transplant recipients [7]; in HIV-1-infected adults, prophylaxis with high-dose valacyclovir resulted in a 33% risk reduction of CMV disease compared to acyclovir [8], [9]. We hypothesized that maternal valacyclovir could reduce CMV transmission by reducing maternal CMV replication both antenatally to prevent *in utero* transmission, and postnatally by reducing breast milk CMV. We further hypothesized that small reductions in maternal CMV replication, as expected with this low dose of valacyclovir, could delay infant acquisition, an important consideration since infants acquire CMV at very young ages. Our final hypothesis was that valacyclovir converts to acyclovir and is present in breast milk in low levels, and might therefore prophylax the infant. Our aims were to measure the impact of maternal valacyclovir on maternal CMV levels, especially in breast milk, and on the rate of infant CMV acquisition.

## Methods

### Ethics Statement

All participants provided written informed consent; mothers provided written consent for both themselves and their child; the study was approved by ethical review committees at the University of Washington and Kenyatta National Hospital.

### Study Design

This study was a secondary aim of a randomized double-blind, placebo-controlled clinical trial (RCT) evaluating the effect of valacyclovir prophylaxis (500 mg twice daily versus placebo) on maternal HIV-1 RNA levels; the study design, methods and main findings have been published (http://clinicaltrials.gov NCT00530777) [10]. An off-site, independent statistician generated random sequentially-numbered study identifiers using a 1∶1 allocation scheme with block sized of 20. Participants were sequentially enrolled and no staff at the study site had knowledge of any participant allocation. The original trial protocol and CONSORT checklist are attached as supplementary information. (Protocol S1, Checklist S1).

Pregnant women were recruited in Nairobi from April 2008 to June 2009; inclusion criteria were seropositive for HIV-1 and HSV-2, CD4 count >250 cells/µl, WHO stage 1 or 2, and ≤34 weeks gestation; women qualifying for HIV-1 treatment were excluded. Participants were randomized at 34 weeks gestation and continued taking valacyclovir or placebo through 12 months postpartum. Samples were collected at 34 and 38 weeks gestation (maternal plasma and cervical swabs) and postpartum at 2, 6, and 14 weeks and 6, 9, and 12 months (maternal plasma and breast milk; infant dried blood spots (DBS) from heel-prick). Infants received standard care and immunizations, and were tested for HIV DNA at 6 weeks, 6 months and 1 year, with additional testing of earlier samples if HIV was detected. Women were counseled to breastfeed exclusively for 6 months, and received prevention of mother-to-child transmission of HIV-1 (PMTCT) prophylaxis according to contemporaneous Kenyan guidelines (maternal zidovudine from 28 weeks, maternal and infant single-dose nevirapine, and 6 weeks of infant zidovudine prophylaxis).

### Laboratory Methods

For maternal blood, breast milk, and cervical specimens, DNA was extracted from 200 µl of maternal plasma, breast milk supernatant or cervical fluid extract using the Qiagen UltraSens kit (Qiagen, California). Blood was extracted from 3×6 mm infant DBS using the QIAsymphony DNA minikit. Real-time quantitative PCR was used to detect the CMV glycoprotein B gene [11]. CMV levels in plasma, breast milk and cervical fluid were reported as DNA copies/ml with a lower limit of detection (LLD) of 100 copies/ml. CMV levels in cervical fluid were adjusted for a concentration factor at extraction, ensuring comparability. CMV DNA levels from DBS were normalized to copies/million cells against a β-globin standard [12] with a LLD of 100 copies/million cells.

### Statistical Methods

Assuming a cumulative CMV incidence in Kenyan infants of 94% at 1 year, a sample size of 74 infants per arm had 90% power to detect 50% reduction in CMV acquisition between arms (α = 0.05, 2-sided test). This sample also provided 80% power to detect a minimum difference of breast milk CMV DNA of 0.5 log_10_ copies/ml between arms. Power calculations assumed up to 15% attrition of mother-baby pairs.

Statistical analyses were conducted as intent-to-treat, using Stata SE v11. All p-values represent 2-tailed tests with alpha = 0.05. For infants, Kaplan-Meier survival analyses and log-rank tests were used to compare time to CMV DNA detection and duration of viremia between arms. CMV infection was defined as the first detection of CMV DNA; CMV clearance was defined as the first of two consecutive negative tests following a positive test.

Viral levels were log_10_-transformed, and levels below the LLD were assigned a value of half the LLD. Fisher’s exact test was used to compare proportions, and the independent T test or Mann-Whitney U test was used to compare means between groups. Generalized estimating equations (GEE) with robust standard errors and exchangeable correlation matrix were used to measure the change in cervical CMV over time; the binomial link function was used to analyze cervical CMV DNA detection as a dichotomized outcome and a linear model with Gaussian distribution was used to analyze CMV level as a continuous outcome.

## Results

### Patient Characteristics

**Figure 1:**
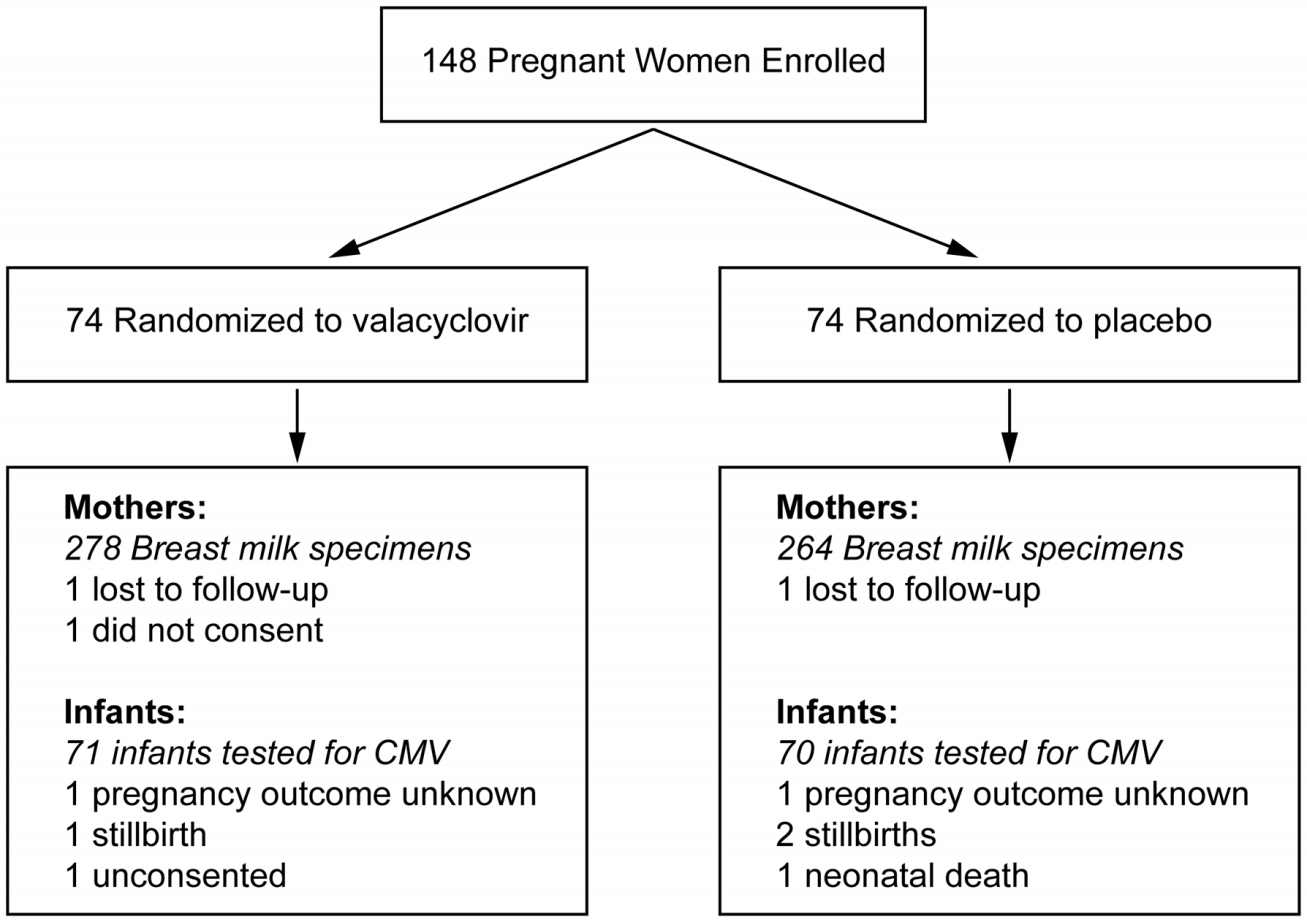
Study flow chart.

From the trial, 147 women consented to CMV testing; a total of 141 infants underwent CMV testing (**Figure 1**). Ten infants acquired HIV-1, of whom 2 later died; 94% of women completed 12 months of follow-up. All mothers received PMTCT. Baseline maternal characteristics were similar between arms (**Table 1** and [10]).

**Table 1:** Maternal and Infant Characteristics.

	Median (IQR) or N (%)	
	placebo	valacyclovir
*Maternal characteristics (N = 147)*	74	73
Plasma CMV tested at 34 weeks	74 (100%)	73 (100%)
Cervical CMV tested at 34 weeks	74 (100%)	73 (100%)
Cervical CMV tested at 38 weeks	49 (66%)	50 (68%)
Breast milk CMV tested	71 (96%)	72 (99%)
No. of visits breast milk CMV tested	4 (3–4)	4 (4–4)
Maternal deaths	2 (2.7%)	1 (1.4%)
*Infant characteristics (N = 141)*	70	71
Follow-up time in days	364 (356–368)	365 (357–369)
Visits tested for CMV	8 (7–8)	8 (7–8)
Acquired HIV-1	4 (5.7%)	6 (8.5%)
Acquired CMV	46 (66%)	47 (66%)
Infant deaths	9 (12%)	3 (4.1%)

### Infant CMV Acquisition

Of infants tested at birth, twice as many infants had detectable CMV in the placebo arm (4/58, 6.9%) compared to the valacyclovir arm (2/61, 3.3%), but this was not statistically significant (p = 0.4). None of these infants had signs or symptoms of congenital CMV. At 2 weeks, 6/68 infants (8.8%) in the placebo arm and 5/70 infants (7.1%) in the valacyclovir arm had detectable CMV DNA (p = 0.8).

**Figure 2:**
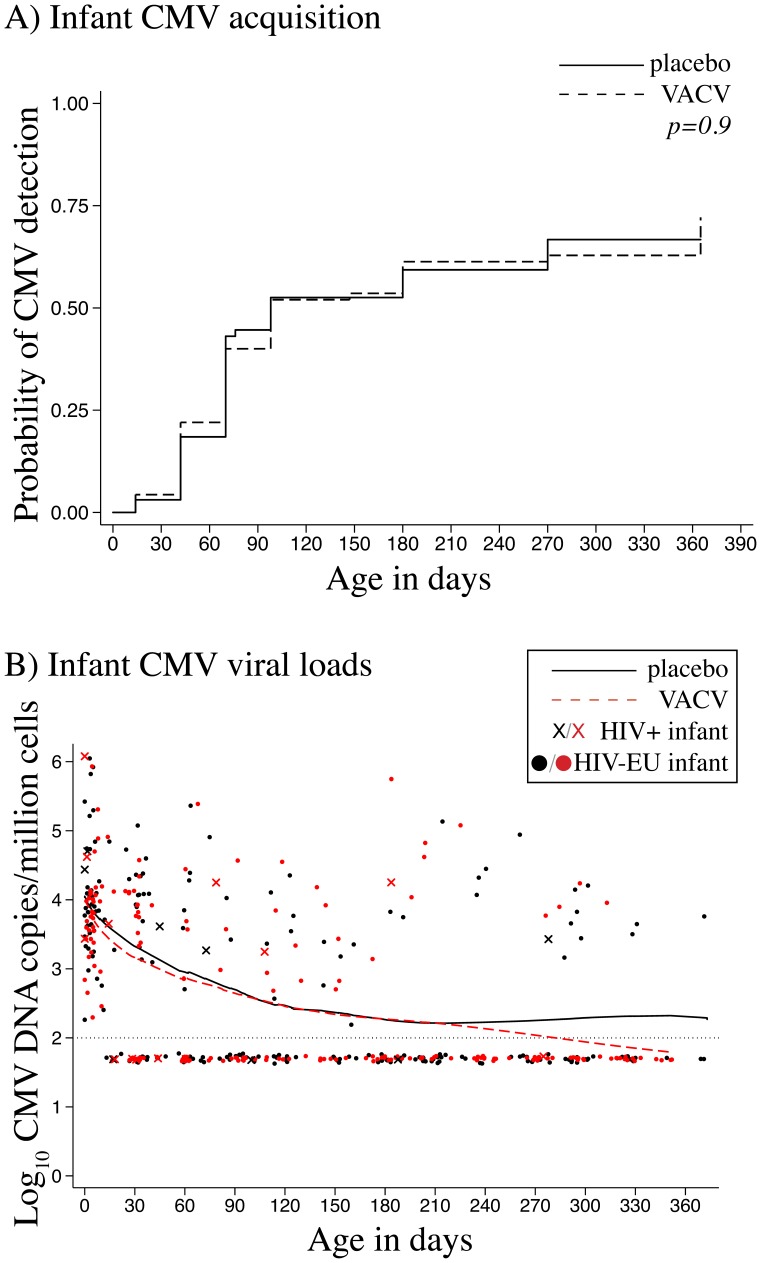
Valacyclovir and infant CMV acquisition. A) Kaplan-Meier survival curves show cumulative probability of CMV DNA detection in infant dried blood spots (DBS) in all infants by randomization arm. P value for log-rank test. B) Loess curves fitted to mean CMV DNA level. HIV-infected infant CMV DNA levels are indicated by Xs, HIV-exposed uninfected infant CMV DNA levels are indicated by closed circles; black indicates placebo and red valacyclovir arm. Dotted line indicates assay limit of detection (100 copies/million cells).

In the placebo arm, a cumulative total of 46/70 infants (66%) had CMV detected by 1 year, compared to 47/71 infants (66%) in the valacyclovir arm (p = 1.0). Median time to CMV detection did not differ between arms; 98 days in the placebo (95%CI = 70−270) and 98 days in the valacyclovir arm (95%CI = 70−270) (**Figure 2A**). When excluding HIV-1-infected infants (6 of whom acquired CMV), there remained no differences in CMV acquisition between arms (p = 1.0, data not shown).

### Infant CMV DNA Levels and Duration of CMV Viremia

Infant CMV DNA levels are shown in **Figure 2B**. Among HIV-1-exposed, uninfected (HIV-EU) infants, CMV DNA levels ranged between 2.2–6.1 log_10_ copies/million cells. There was no difference in mean peak CMV DNA level between placebo (4.2 log_10_ copies/million cells, SE +0.87) and valacyclovir arms (4.1 log_10_ copies/million cells, ±SE = 0.82; p = 0.6), and CMV DNA levels were similar at each visit between arms (data not shown). Among 87 HIV-EU infants who had ≥2 tests following first detection of CMV, the proportion of infants who cleared viremia (p = 0.8), and duration of CMV viremia (p = 0.9) was similar between arms. In the placebo arm, 73% (32/44) of infants cleared CMV at a median of 23 days (95%CI = 15−193), and in the valacyclovir arm, 70% (30/43) of infants cleared CMV at a median of 42 days (95%CI = 30−193). Among the 10 infants who acquired HIV-1, 6 also acquired CMV; their CMV DNA levels ranged between 3.3 −6.1 log_10_ copies/million cells.

### Maternal Plasma and Breast Milk CMV DNA Levels

**Figure 3:**
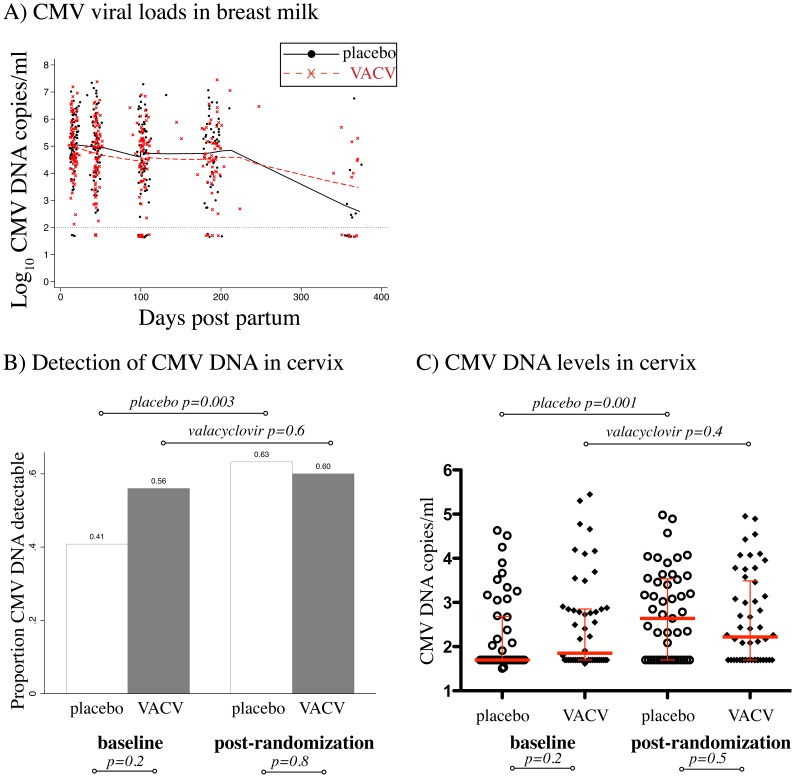
Valacyclovir and maternal CMV levels. A) Loess curves fitted to mean CMV DNA levels in breast milk samples collected at 2, 6 and 14 weeks postpartum, and 6, 9 and 12 months postpartum. Red line and markers indicate women randomized to valacyclovir. Dotted line indicates assay limit of detection (100 copies/ml). Note: few women were breastfeeding after 180 days postpartum. B) Bars show proportion of women with detectable CMV DNA in the cervix at pre-randomization (34 weeks gestation) and 4 weeks post-randomization (38 weeks gestation). P values below the graph compare the proportion of detectable responses between arms, p values above graph compare the proportion of detectables within each arm, over the two time-points. C) Plots show individual CMV DNA levels for women at baseline and post-randomization, red middle bars show group medians, red whiskers show upper and lower quartiles. P values below the graph show comparison of CMV DNA levels between arms, p values above graph compare CMV DNA levels within each arm, over the two time-points.

Pre-randomization, only 7/145 (4.8%) women had detectable CMV in their plasma, and we lacked power to compare study arms. Of 143 women who provided breast milk, CMV DNA was detected in 142 women (99%), and in 499/542 of their specimens (92%). At each time-point, there was no significant difference in the mean quantity of CMV DNA between arms (p>0.05, all comparisons, data not shown). The mean peak CMV viral load in breast milk was also similar between placebo (5.7 log_10_ copies/ml ±SD = 0.99) and valacyclovir arms (5.4 log_10_ copies/ml ±SD = 1.0, p = 0.2). In both arms, mean breast milk CMV levels declined over time, with the highest levels noted at 2 weeks postpartum. (**Figure 3A**).

### Maternal Cervical CMV Shedding

Among 99 women with serial sampling of cervical secretions, the proportion with detectable CMV DNA increased between baseline and 38 weeks in the placebo arm (from 41% to 63%; p = 0.003), but did not change in the valacyclovir arm, (from 56% to 60%, p = 0.6; **Figure 3B**). Similarly, in the placebo arm CMV DNA levels increased significantly over time (baseline median = 1.7 log_10_ copies/ml, IQR = 1.7−2.7 log_10_ copies/ml, 38 week median = 2.6 log_10_ copies/ml, IQR = 1.7−3.5 log_10_ copies/ml; p = 0.001) but did not change significantly in the valacyclovir arm (baseline median 1.9 log_10_ copies/ml, IQR = 1.7−2.8 log_10_ copies/ml, 38 week median = 2.2 log_10_ copies/ml, IQR = 1.7−3.5 log_10_ copies/ml; p = 0.4, **Figure 3C**).

## Discussion

In this RCT of valacyclovir dosed for HSV-2 suppression, we found that valacyclovir at a dose of 500 mg twice daily had a modest effect on the pattern of maternal cervical CMV shedding, but had no effect on maternal breast milk CMV DNA levels or infant CMV acquisition and viremia. To our knowledge, this is the first RCT evaluating the effect of maternal antiviral prophylaxis to prevent CMV transmission to children. Including these secondary endpoints into an ongoing RCT enabled rapid evaluation of the potential benefit of an affordable, well-tolerated drug at doses routinely used in pregnancy, and allowed us to evaluate this intervention in a population of HIV-1-exposed infants in Kenya who are vulnerable to early CMV infection.

Detection of early CMV infection in 7% of infants by 2 weeks of age confirms high prevalence of early infant acquisition of CMV found in an earlier cohort studied in Kenya, where 6% of HIV-1-seronegative infants born to HIV-1-seropositive mothers had CMV detected at birth and 20% detected by one month [2]. Lower prevalences of early infant CMV have been observed among HIV-1-seropositive mothers who start triple antiretroviral therapy before or during pregnancy: 3.6% of infants born to HIV-1 infected mothers had CMV detected at birth in a US cohort [13] and 1.2% of similar infants had CMV detected at birth in the French ANRS cohort [14]. This high prevalence of early infant CMV infection in our cohort, where women received zidovudine monotherapy and perinatal nevirapine, suggests that HIV-exposed infants are at risk of early acquisition of CMV due to maternal factors such as immunosuppression. In this cohort, mothers had T cell counts >250 cells/µl but had only a few months of exposure to zidovudine at delivery. One mechanism to explain the high prevalence of infant CMV may be the observation that HIV-1 infected mothers may transfer less protective antibody to their infants perinatally [15].

Valacyclovir treatment was previously shown in this cohort to reduce maternal HIV-1 RNA levels by ∼0.5 log_10_ copies/ml in plasma and breast milk [10]. Previous studies report correlations between CMV and HIV-1 levels in plasma and breast milk [2], [16]. HIV-induced immunosuppression impairs CMV containment, and *in vitro* studies have demonstrated bi-directional cellular interactions between the two viruses that reciprocally enable infectivity and replication [17]. In light of this synergy, reductions in breast milk CMV DNA secondary to HIV-1 RNA reduction were expected, but were not observed.

The dose of valacyclovir was inadequate to impact breast milk CMV DNA levels, even though valacyclovir penetrates the breast compartment. Prophylaxis studies against CMV disease in organ transplant patients have used much higher doses of acyclovir (1.5–8 g/day) [7], [18] with significant effect. A pilot study reported using 8 g of valacyclovir in pregnancy [19] to treat mothers of fetuses with congenital CMV infection. Controlled trials in pregnancy or breastfeeding with acyclovir or novel antivirals have not been conducted. Despite the limitations of 500 mg twice-daily dosing of valacyclovir in preventing CMV reactivation, our hypothesis was that our more realistic goal of small reductions in the high CMV load in breast milk would translate into delays in infant acquisition. These delays may be significant: in newborn infants living in areas of high infant mortality, delaying CMV acquisition by even 2–4 weeks could translate into morbidity and mortality benefits. Our trial results, however, showed conclusively that this dose of valacyclovir did not benefit HIV-1 exposed infants.

We found a modest effect of valacyclovir on maternal cervical CMV shedding. In a prior study in the Gambia, maternal genital shedding of CMV was associated with both congenital and early infant infections [5]. Few studies have examined effects of antiviral therapy on CMV levels in the genital tract; an RCT in HIV-1-infected men found no effect of 500 mg twice daily valacyclovir on semen CMV levels [20]. In our placebo arm, cervical CMV shedding increased between 34 and 38 weeks, consistent with reports of increased shedding during late pregnancy [21], but this increase was attenuated in the valacyclovir arm. CMV shedding has been observed to increase during the luteal phase of the menstrual cycle, and is associated with progesterone and estradiol levels [22], suggesting hormonal cervical changes may be determinants of local CMV replication. The genital tract was the only compartment where we saw a difference in CMV shedding, which may be due to valacyclovir’s penetration to cervical and vaginal tissues.

Strengths of this study include its prospective randomized design and robust power to detect differences in infant acquisition and breast milk CMV levels. Women had high adherence to the study protocol, verified by valacyclovir detection in breast milk [23] and HIV-1 suppression. Because no infant blood was obtained, we were unable to confirm CMV infection using serology, and we may underestimate the true number of infant infections, particularly those with low-level or short-duration viremia. Additionally, comparison of plasma and DBS specimens demonstrates lower sensitivity of DBS with low CMV levels (Atkinson, in preparation). However, even if some infant CMV infections were missed, the 93 transmission events observed were adequate to detect a significant impact on CMV transmission, and our randomized design preserves our ability to discern effects even with possible underascertainment. We are unable to comment on the effects of valacyclovir on HIV-1-seronegative women or HSV-2- seronegative coinfected women and their infants.

In conclusion, maternal valacyclovir had a modest impact on cervical CMV shedding in late pregnancy, but did not affect infant CMV acquisition or breast milk CMV levels. Early CMV infection may be one of a series of concurrent infections that adversely affect infant health, growth and survival in developing countries, especially among HIV-1-exposed infants. Maternal antiretroviral therapy has reduced infant CMV infection among HIV-1-exposed infants in developed countries, but may not be as effective in developing countries, where most CMV acquisition occurs in the first year of life, and where breastfeeding continues to be recommended for infants of HIV-1-seropositive mothers. Maternal antiviral prophylaxis with higher doses of valacyclovir or novel antiviral agents to prevent or delay infant CMV acquisition in the early postnatal period may warrant further study.

## Supporting Information

Checklist S1 (DOC)

Protocol S1 (DOC)
