# Supplementary material for: Maternal Valacyclovir and Infant Cytomegalovirus Acquisition: A Randomized Controlled Trial among HIV-Infected Women
Source: PLoS One. 2014 Feb 4;9(2):e87855. doi: 10.1371/journal.pone.0087855 (PMC3913686; doi:10.1371/journal.pone.0087855)
Supplement: Protocol S1 — (DOC) [file pone.0087855.s002.doc]

**Valacyclovir and vertical cytomegalovirus transmission-PROTOCOL**

**Protocol Team Roster**

| **Investigator** | **Role on Project** | **Institutional Address** | **Signature** |
| --- | --- | --- | --- |
| Jennifer Slyker, PhD | Principal Investigator | University of Washington, Department of Medicine, Box 359931, Seattle, WA 98104 |  |
| James Kiarie, MBChB.,  MMed, MPH | Principal Investigator | University of Nairobi, Department of Obstetrics and Gynaecology, PO Box 19676, University of Nairobi, Nairobi, Kenya |  |
| Carey Farquhar, MD, MPH | Co-investigator | University of Washington, Department of Medicine and Epidemiology, Division of Allergy and Infectious Diseases  c/o IARTP, Box 359909, Seattle, WA 98104 |  |
| Barbra Richardson, PhD | Co-investigator | University of Washington, Department of Biostatistics  c/o IARTP, Box 359909, Seattle, WA 98104 |  |
| Grace John-Stewart, MD, MPH, PhD | Co-investigator | University of Washington, Department of Medicine and Epidemiology,  c/o IARTP, Box 359909, Seattle, WA 98104 |  |
| Dorothy Mbori-Ngacha, MBChB, MMed, MPH | Co-investigator | University of Nairobi, Department of Paediatrics and Child Health  PO Box 19676, University of Nairobi, Nairobi, Kenya, |  |
| Alison Drake, MPH | Co-investigator | University of Washington, Department of Medicine and Epidemiology,  c/o IARTP, Box 359909, Seattle, WA 98104 |  |
| Francisca Ongecha-Owuor, MBChB, MMed | Co-investigator | University of Nairobi, Department of Obstetrics and Gynaecology  P.O.Box 34646-00100, University of Nairobi, Kenya |  |
| Alison C. Roxby, MD, MSc | Co-investigator | University of Washington, Infectious Diseases Fellow  c/o IARTP, Box 359909, Seattle, WA 98104 |  |

**A. SUMMARY**

Half a million infants acquired HIV-1 last year, and the development of strategies to improve the health of HIV-infected children is of critical importance. Cytomegalovirus (CMV) contributes significantly to morbidity and mortality in HIV-1 infected infants. We propose a nested randomised controlled trial (RCT) to evaluate the effect of valacyclovir during pregnancy and breastfeeding on vertical transmission of CMV. We hypothesise valacyclovir will reduce maternal CMV levels in blood, breastmilk and the cervix and reduce vertical CMV transmission. The project will utilise stored specimens from an ongoing RCT. A total of 148 HIV-1/HSV-2 co-infected pregnant women with CD4>250 cells/μl will be enrolled from Nairobi City Council Clinics. Enrollment is ongoing and expected to complete at the end of 2009. Women are randomized to either valacyclovir suppressive therapy or placebo during pregnancy and mother-infant pairs will be followed for 12 months postpartum. Follow-up visits will be scheduled at 38 weeks gestation; birth; 2, 6, 10 and 14 weeks; and 6, 9, and 12 months postpartum. Maternal blood, genital swabs, and breastmilk will be used to determine the effect of valacyclovir on CMV DNA levels. Infant filter papers collected at birth; 2, 6, 10 and 14 weeks; and 6, 9, and 12 months will be used to diagnose infant CMV. If valacyclovir significantly reduces the rate of CMV transmission, this strategy may offer a supplemental intervention to reduce mortality in HIV-infected infants.

**B. BACKGROUND**

B1. Introduction: The epidemiology of CMV in HIV-infected infants

Cytomegalovirus (CMV) is the most common viral cause of congenital disease globally, affecting 0.2-3% of live births in higher-income nations, with rates from high as 4-14% reported in Africa (1-3). Among healthy newborns, congenital cytomegalovirus (CMV) infection may result in severe disability, including sensorineural hearing loss and mental retardation (4, 5). CMV prevalence varies with socioeconomic conditions (6); poor communities have a relatively higher prevalence and an earlier incidence of infection. A recent survey reported 54% of American adults in their thirties to be CMV seropositive (7), whereas >85% of Kenyan infants acquire CMV before they are 3 months old (manuscript in preparation, (8)). Vertical CMV transmission can occur *in utero*, intrapartum, or via breastmilk. Several studies have examined correlates of CMV transmission. The detection of CMV DNA in the female genital tract (3) and breastmilk (9, 10), is associated with vertical transmission; but the detection of CMV DNA in the blood is not (3, 11).

In the HIV-1 infected infant, CMV causes significant morbidity and mortality. In addition to the typical sequelae that arise from congenital infections, both congenital and postnatally acquired CMV can cause gastrointestinal disease, failure to thrive, hepatomegaly and splenomegaly in the HIV-1 infected infant. In US and European studies, co-infection with CMV has been noted in ~40% of HIV-1 infected infants during the first year of life and has been associated with a ~2.5-fold increased risk of disease progression (12-14). A recent study conducted in Kenya found that the detection of CMV DNA in the blood of HIV-1 infected women at delivery was associated with a 3 to 4-fold increased risk of mortality among their infants, after adjusting for the effects of maternal CD4 or viral load (15). This observation may be explained by earlier CMV transmission from the CMV-DNA-positive women; co-infection with HIV-1 and CMV before 1 month of age was associated with increased mortality compared to infants with later co-infection (manuscript in preparation, (8)).

B2. Rationale for limited maternal antiviral therapy to interrupt vertical CMV transmission

Together these data suggest that preventing or delaying CMV co-infection may improve survival in HIV-1 infected infants. No CMV vaccine is yet available, and immunization is likely to be of limited use in the prevention of *in utero* or intrapartum transmission from CMV seropositive women. Replacement feeding may prevent or delay breastmilk CMV transmission but confers additional risks to the infant. No clinical trials have yet been conducted which evaluate CMV suppression in the mother to reduce the risk of vertical CMV transmission. Valacyclovir is currently the only antiviral available that inhibits CMV replication and is approved for use in pregnant and breastfeeding women. Ganciclovir is more effective than valacyclovir in inhibiting CMV replication, but is not recommended for use in pregnant and breastfeeding women due to toxicities and teratogenicity observed during animal testing (16).

Valacyclovir is used effectively to prevent CMV infection and CMV disease in transplant recipients (17-19). One trial has been published reporting valacyclovir prophylaxis against CMV in subjects with advanced HIV-1 infection (20, 21). In HIV-1 infected subjects with CD4 counts below 100mm^3^, treatment with 2 g q.i.d. valacyclovir prophylaxis resulted in a 33% risk reduction of CMV disease compared to acyclovir (21) and the maintenance of a 1.3 log reduction in CMV viral load in blood over a 16 week period (20). However, significant toxicities were observed in the patients receiving high-dose valacyclovir and a trend toward increased mortality was observed in this group in comparison to subjects receiving acyclovir (21). A subsequent trial in renal transplant recipients investigated the efficacy of a lower dose of valacyclovir (1 g t.i.d.) adjusted to creatinine clearance, and found a 4-fold reduction in CMV disease at 6 months (22). These data suggest that although valacyclovir prophylaxis may be effective for the prevention of CMV infection and disease, further studies are needed to establish the lowest effective dose and minimize side effects. The current RCT is providing valacyclovir at 500 mg b.i.d., and this is the current recommended dose for the prevention of recurrent outbreaks of genital herpes. It is unknown what effect this dose will have upon CMV replication in the blood, genital tract or breastmilk.

Antiretroviral therapy has been used effectively in the prevention of mother-to-child transmission of HIV-1. We propose that antiviral therapy may be similarly effective in reducing the risk of CMV transmission. A lower dose of valacyclovir than that needed for CMV treatment/prophylaxis may inhibit CMV replication sufficiently in the mother to prevent transmission to the infant. The pharmacokinetics of valacyclovir may increase drug concentrations at sites of vertical CMV transmission; in pregnant women treated with valacyclovir, acyclovir was found to be concentrated in the amniotic fluid (~2x the plasma concentration (23)). During treatment with acyclovir, the drug is concentrated in the breastmilk (3-4x the plasma concentration (24, 25)); since valacyclovir is metabolized into acyclovir, a similar concentrating effect is expected during treatment with valacyclovir.

B3. Significance

We hypothesize that women treated with valacyclovir for the suppression of HSV-2 will also experience a reduction in CMV viral load, and that this will reduce the risk of late intrauterine and intrapartum CMV transmission, and delay breastmilk CMV transmission. We propose this pilot study to collect data to support a large randomized clinical trial of maternal CMV suppression to reduce CMV acquisition and mortality in HIV-infected infants. If successful, this strategy could offer a low-cost, feasible intervention that if added to current mother-to-child HIV-1 prevention strategies, could improve the prognosis of HIV-infected infants in Kenya.

**C. SPECIFIC AIMS AND HYPOTHESES**

We hypothesise that treatment with valacyclovir during pregnancy and breastfeeding will result in the reduction of CMV viral load in the female genital tract and breastmilk, and will reduce the risk of vertical CMV transmission. We will nest this study into an ongoing randomized clinical trail providing twice-daily valacyclovir or placebo to a cohort of HIV-1 infected pregnant women living in Nairobi, Kenya. Infant CMV infection will be defined as the first of two positive sequential CMV tests.

Specific Aim 1: To compare the risk of vertical CMV transmission during 12 months of follow-up between women randomized to valacyclovir or placebo.

Specific Aim 2: To compare CMV viral load in women receiving valacyclovir vs placebo. We will study viral loads in the blood, genital tract and breastmilk.

**D. STUDY DESIGN**

D1. Overview

As part of the ongoing RCT, HIV-1-seropositive pregnant women with CD4>250 cells/μl attending an antenatal care clinic in Nairobi, Kenya will be screened for HSV-2 seropositivity prior to 32 weeks gestation and 148 women who meet the eligibility criteria detailed in D.3. will be randomized to receive either valacyclovir suppressive therapy or placebo beginning at 34 weeks gestation. All women will receive the standard of care per Kenyan national guidelines for prevention of mother-to-child HIV-1 transmission among women with CD4>250 cells/μl. Maternal blood specimens and genital swabs will be collected during antenatal visits and breast milk will be collected during postpartum follow-up for HIV-1 RNA assays. Infants will have a blood specimen taken within 2 days of birth; at 2, 6, 10, and 14 weeks; and at 6, 9, and 12 months of age for HIV-1 DNA assays. Maternal blood at 34 and 38 weeks gestation, will be used to assess creatinine, and infant blood collected at 6 weeks will be used to determine infant creatinine and liver function. This nested study proposes to add testing for CMV viral load in stored specimens collected from the parent study.

D2. Study site

The ongoing double blind, placebo-controlled randomized clinical trial will enroll 148 HIV-1/HSV-2 co-infected pregnant women seeking antenatal care at the Mathare North City Council Clinic in Nairobi, Kenya. Referrals will be accepted from women seeking antenatal care at nearby city council clinics.

Currently, the Mathare North Clinic and Pumwani Hospital has a program funded by The President’s Emergency Plan for AIDS Relief (PEPFAR) for prevention of mother-to-child transmission of HIV at this clinic and through this program all HIV-1-infected women will be offered standardized counseling, CD4 testing, and appropriate antiretrovirals based on CD4 count to prevent HIV-1 transmission, regardless of study participation. For women with CD4>250 cells/μl this consists of oral zidovudine (ZDV) 300 mg beginning at 28 weeks gestation, oral ZDV 300 mg at the onset of labor and every 3 hours until delivery, and a single dose of nevirapine (NVP) 200 mg at the onset of labor for the mother. The infant receives NVP 2 mg/kg oral suspension immediately after birth plus ZDV 4mg/kg twice daily for 28 days. As with our other studies, we will integrate the proposed clinical trial into the existing infrastructure of this program, and modify PMTCT procedures if guidelines are updated in Kenya.

D3. Screening and enrollment

Women seeking antenatal care prior to 32 weeks gestation at the Mathare North Clinic, Pumwani Hospital or nearby City Council Clinics will be asked to consent to screening for the study. Those who accept will have blood drawn for HSV-2 serology and be asked to return in 2 weeks for their results and possible enrollment into the trial. Women who return to the clinic at 34 weeks gestation and meet the eligibility criteria will be asked for consent to enroll in the study. Eligibility criteria for the proposed study include the following:

- HIV-1 seropositive
- HSV-2 seropositive
- Plans to deliver in Nairobi
- Resides and plans to remain in Nairobi for 12 months postpartum
- 18 years of age or older
- CD4 count>250 cells/μl
- No indication for highly active antiretroviral therapy (e.g., WHO stage III or IV)
- No known hypersensitivity to valacyclovir or acyclovir.

At the enrollment visit, women who have consented will be administered a questionnaire, undergo physical examination and specimen collection, and will be randomized to either the valacyclovir or placebo arm of the study. Women in both arms will receive standard antiretroviral prophylaxis, as described above, a daily multivitamin, syphilis treatment if indicated, and syndromic management for reproductive tract infections per national guidelines. Questions will be asked pertaining to demographic characteristics, medical history, and sexual history, and blood will be drawn for HIV-1 RNA viral load, syphilis testing, immune activation markers, and baseline creatinine. At the end of the enrollment visit, women will be randomized to receive either 500 mg valacyclovir twice daily or placebo twice daily based on computer-generated block randomization. All study investigators will be blinded to which arm study participants have been randomized and statistical analyses will be conducted with blinding intact.

D4. Follow-up visits

One antenatal follow-up visit will be scheduled at 38 weeks gestation. During the follow-up visit at 38 weeks gestation, the same procedures will be followed as at enrollment for questionnaire administration, physical examination, and blood collection for HIV-1 RNA and creatinine. Postpartum follow-up visits will be scheduled near delivery; at 2, 6, 10, and 14 weeks; and 6, 9, and 12 months postpartum. All visits, except the 2 week postpartum visits, follow routine antenatal care and infant immunization schedules. In accordance with the first scheduled infant immunization, women who deliver outside the study clinic will be asked to return to the clinic with their infant within 2 days postpartum. At this visit, or immediately after birth for women who deliver at the study clinic, we will provide infant feeding counseling and collect an infant filter paper blood specimen using a heel prick for determination of infant HIV-1 and CMV status. Additional infant filter paper blood specimens will be collected at 2, 6, 10, and 14 weeks and 6, 9, and 12 months of age to determine timing of HIV-1 and CMV infection. Maternal blood and breast milk specimens will be taken at 2, 6, and 14 weeks and 6 and 12 months postpartum for HIV-1 RNA and CMV DNA viral load. At 12 months postpartum all women will stop taking either the valacyclovir suppressive therapy or placebo. In Figure 1, we have included details on procedures at enrollment and scheduled follow-up visits.

**Figure 1. Study visits and specimen collection**

**Women receiving antenatal care at Mathare North City Clinic or Pumwani Hospital**

**Women receiving antenatal care at Mathare North City Clinic or Pumwani Hospital**

**Women receiving antenatal care at Mathare North City Clinic or Pumwani Hospital**

Maternal blood

Maternal blood, cervical/genital swabs

Maternal blood, cervical/genital swabs

Maternal blood and breast milk (2, 6 and 14 weeks; 6 and 12 months)

Infant blood (2, 6, 10, and 14 weeks; 6, 9 and 12 months)

Infant serum (6 weeks)

**Specimens obtained**

**Screening**

≤ 32 weeks gestation

**Enrollment and Randomization**

34 weeks gestation

**Antenatal Follow-up**

Bimonthly; Specimens at 38 weeks

**Delivery**

**Postpartum Follow-up**

≤ 2 days; 2, 6, 10, and 14 weeks;

6, 9, 12 months

D5. Laboratory Procedures

Specimens and testing procedures are summarised in Table 1.

*Procedures ongoing as part of the parent RCT*

Blood samples obtained at the screening visit will be used to determine syphilis serostatus by rapid plasma reagin (RPR) (Becton Dickinson and Company), confirm syphilis serostatus using the *Treponema pallidum* hemagglutination assay (Randox Laboratories Ltd), confirm HIV-1 serostatus, and determine maternal HSV-2 serostatus by the Focus ELISA (HerpeSelect HSV-2 ELISA; Focus Diagnostics) ^(26)^ in the University of Nairobi’s Clinical Trials Laboratory. CD4 T cell counts will be performed on maternal blood specimens collected at the screening and 6 month and 12 month postpartum visits using flow cytometry (FACSCaliber, Becton Dickinson and Company). Immune activation markers will be measured from women at study entry, 6 months and 12 months using flow cytometry (FACSCaliber, Becton Dickinson and Co.). Infant filter paper specimens will be assayed for HIV-1 DNA using polymerase chain reaction (PCR) at the Centers for Disease Control (CDC) / Kenyan Medical Research Institute (KEMRI). Creatinine and ALT testing will be performed at the University of Nairobi Paediatrics Department Laboratory.

Plasma, cervical, and breast milk specimens will be processed in the Clinical Trials Laboratory, cryopreserved and stored at -80°C prior to being shipped to Seattle for testing. All maternal HIV-1 RNA assays from blood, cervical, and breast milk specimens will be conducted in Seattle at the Fred Hutchinson Cancer Research Center in Dr. Julie Overbaugh’s laboratory. HIV-1 RNA assays will be performed as previously described.^(27)^ Genital specimens for HSV-2 DNA assays will be processed in the Clinical Trials Laboratory and stored at -20°C prior to being shipped to Seattle for testing The University of Washington Clinical Virology Laboratory at the University of Washington’s Virology Division will perform the HSV-2 DNA PCR assays, as described elsewhere.(28, 29) In Table 1, we have included details on laboratory tests at enrollment and scheduled follow-up visits.

*New procedures proposed for the nested CMV study*

CMV diagnosis of infant specimens will be made from dried blood spots collected onto filter paper. Following HIV-1 diagnostics at the Kenya Medical Research Institute, the specimens will be returned to the study laboratory for CMV testing. CMV diagnosis will be performed with nested PCR performed in triplicate as described by Barbi et al (30). CMV viral loads will be measured in cryopreserved plasma, cervical swabs, and breastmilk specimens. CMV DNA will be isolated in the Nairobi study laboratory using the Qiagen UltraSens virus extraction kit (Qiagen, Valencia, California, USA). Extracted (non-infectious) DNA will be shipped to our collaborating laboratory at University College London for viral load quantification using real-time PCR detection of the glycoprotein B gene (31). We have decided to utilize this specific laboratory because they have developed and patented a protocol specifically for the determination of CMV viral loads.

| **Table 1. Clinical testing and laboratory assays performed at baseline and during follow-up** | | | | | | | | | | | |
| --- | --- | --- | --- | --- | --- | --- | --- | --- | --- | --- | --- |
|  | **Screening** | **Enrollment and**  **antenatal follow-up** | | **Postpartum Follow-up** | | | | | | | |
|  | **≤ 32** | **34** | **38** | **≤ 2** | **2** | **6** | **10** | **14** | **6** | **9** | **12** |
|  | **weeks** | | | **days** | **weeks** | | | | **months** | | |
| **Maternal tests or assays** |  |  |  |  |  |  |  |  |  |  |  |
| Rapid HIV testing with ELISA confirmation | **X** |  |  |  |  |  |  |  |  |  |  |
| HSV-2 serology | **X** |  |  |  |  |  |  |  |  |  |  |
| RPR and TPHA for syphilis |  | **X** |  |  |  |  |  |  |  |  |  |
| Serum creatinine |  | **X** | **X** |  |  |  |  |  |  |  |  |
| CD4+ T cell count | **X** |  |  |  |  |  |  |  | **X** |  | **X** |
| Immune activation markers (CD 38, HLA-DR) |  | **X** |  |  |  |  |  |  | **X** |  | **X** |
| Plasma HIV RNA PCR |  | **X** | **X** |  | **X** | **X** |  | **X** | **X** |  | **X** |
| Cervical swab HIV RNA PCR |  | **X** | **X** |  |  |  |  |  |  |  |  |
| Breast milk HIV RNA PCR |  |  |  |  | **X** | **X** |  | **X** | **X** |  | **X** |
| Genital HSV-2 DNA PCR |  | **X** | **X** |  |  |  |  |  |  |  |  |
| CMV serology |  | **X** | **X** |  |  |  |  |  |  |  |  |
| Plasma CMV viral load PCR |  | **X** | **X** |  | **X** | **X** |  | **X** | **X** |  | **X** |
| Genital CMV viral load PCR |  | **X** | **X** |  |  |  |  |  |  |  |  |
| Breastmilk CMV viral load PCR |  |  |  |  | **X** | **X** |  | **X** | **X** |  | **X** |
| *Total volume blood (ml)* | **20** | **20** | **20** |  | **20** | **20** |  | **20** | **20** | **20** | **20** |
|  |  |  |  |  |  |  |  |  |  |  |  |
| **Infant tests or assays** |  |  |  |  |  |  |  |  |  |  |  |
| HIV-1 DNA PCR |  |  |  | **X** | **X** | **X** | **X** | **X** | **X** | **X** | **X** |
| Serum creatinine |  |  |  |  |  | **X** |  |  |  |  |  |
| Serum ALT |  |  |  |  |  | **X** |  |  |  |  |  |
| CMV DNA PCR |  |  |  | **X** | **X** | **X** | **X** | **X** | **X** | **X** | **X** |
| CMV viral load |  |  |  | **X** | **X** | **X** | **X** | **X** | **X** | **X** | **X** |
|  |  |  |  |  |  |  |  |  |  |  |  |
| *Total volume blood (ml)* |  |  |  | **3** | **3** | **5** | **3** | **3** | **3** | **3** | **3** |

NOTE. All specimens and tests are shown, proposed CMV testing is shown shaded in grey.

D6. Timeline

Enrollment is ongoing and should be completed by the end of 2009. We expect to begin CMV PCR assays this year and to complete them in approximately 1 year’s time.

Table 2. Study timeline

| **Year 1** | Completion of enrollment and follow-up, begin PCR for CMV viral load |
| --- | --- |
| **Year 2** | Complete CMV PCRs, data analysis and preparation of manuscripts |

D7. QA/QC PROCEDURES

Internal laboratory quality control procedures will be put into place, according to standard protocols.

**E. DATA ANALYSIS AND SAMPLE SIZE CALCULATIONS**

E.1. The primary analyses for each aim will be conducted using the intent-to-treat principle and baseline parameters in the 2 arms will be compared to determine adequacy of randomization.

Specific Aim 1: To compare the risk of vertical CMV transmission during 12 months of follow-up between women randomized to valacyclovir or placebo.

Survival analysis will be used to compare the time to infant CMV acquisition between mother-infant groups randomized to valacyclovir during pregnancy and breastfeeding versus placebo.

Specific Aim 2: To compare CMV viral load in women receiving valacyclovir vs placebo. We will study viral loads in the blood, genital tract and breastmilk.

CMV viral load in maternal breastmilk, genital swabs, and plasma will be compared between HIV-infected women randomized to valacyclovir suppressive therapy versus placebo, using the T test or Mann-Whitney U test.

E.2. Sample Size Calculations:

Aim 1:

Kaplan-Meier survival estimates will be used to determine the timing of CMV transmission in mother-infant pairs receiving valacyclovir and placebo. From a previous study conducted by our group in Nairobi (8), we estimate that 94% of infants born to HIV-1 infected women in the control arm will have acquired CMV by 12 months of age, regardless of infant HIV-1 infection status. The study will enroll 148 women. Allowing for 15% attrition, we expect 126 women to complete the study. Assuming a 50% reduction in CMV acquisition in the intervention arm versus the control arm, we expect approximately 90 infants to acquire CMV during the study, giving us 90% power to detect a 50% reduction in CMV transmission risk between the arms with α=0.05 using a 2-sided test.

Aim 2:

Plasma, genital, and breastmilk viral loads will be compared between study groups using independent sample t-tests. There are no currently published data documenting the decrease in CMV viral load in patients receiving twice daily 500 mg valacyclovir. We have thus estimated the log difference we will be powered to detect for various β values and standard deviations within the estimated final sample size of 126 participants (Table). For example with a hypothetical standard deviation of 1.0, we will have 80% power to detect a minimum difference of 0.5 log between study arms at α=0.05.

Table 3. Power calculations.

| **Standard deviation** | **Power** | | |
| --- | --- | --- | --- |
|  | **80%** | **85%** | **90%** |
| 1.0 | 0.50 | 0.54 | 0.58 |
| 1.5 | 0.75 | 0.81 | 0.87 |
| 2.0 | 1.00 | 1.07 | 1.16 |

**F. DATA COLLECTION INSTRUMENTS**

No new data collection instruments are proposed.

**G. STUDY LIMITATION**

The gold standard technique for the diagnosis of CMV infection is the isolation of virus from urine or saliva, followed by culture or PCR. Since the proposed project is nested into an ongoing study nearing completion, it is not possible for us to collect these specimens for analysis. Quantitative PCR of plasma is highly sensitive and specific, and agrees well with urine-based culture and PCR diagnostic methods.

**H. REFERENCES.**

1. Stagno, S., D. W. Reynolds, E. S. Huang, et al. 1977. Congenital cytomegalovirus infection. *N Engl J Med* 296:1254-1258.

2. Bello, C., and H. Whittle. 1991. Cytomegalovirus infection in Gambian mothers and their babies. *J Clin Pathol* 44:366-369.

3. Kaye, S., D. Miles, P. Antoine, et al. 2008. Virological and immunological correlates of mother-to-child transmission of cytomegalovirus in The Gambia. *J Infect Dis* 197:1307-1314.

4. Pass, R. F., S. Stagno, G. J. Myers, et al. 1980. Outcome of symptomatic congenital cytomegalovirus infection: results of long-term longitudinal follow-up. *Pediatrics* 66:758-762.

5. Boppana, S. B., R. F. Pass, W. J. Britt, et al. 1992. Symptomatic congenital cytomegalovirus infection: neonatal morbidity and mortality. *Pediatr Infect Dis J* 11:93-99.

6. Stagno, S., R. F. Pass, G. Cloud, et al. 1986. Primary cytomegalovirus infection in pregnancy. Incidence, transmission to fetus, and clinical outcome. *Jama* 256:1904-1908.

7. Staras, S. A., S. C. Dollard, K. W. Radford, et al. 2006. Seroprevalence of cytomegalovirus infection in the United States, 1988-1994. *Clin Infect Dis* 43:1143-1151.

8. Slyker, J. A., B. L. Lohman-Payne, G. C. John-Stewart, et al. 2008. Primary CMV infection in Kenyan HIV-1 infected infants. In *in preparation* Submitted

9. Vochem, M., K. Hamprecht, G. Jahn, et al. 1998. Transmission of cytomegalovirus to preterm infants through breast milk. *Pediatr Infect Dis J* 17:53-58.

10. Hamprecht, K., J. Maschmann, M. Vochem, et al. 2001. Epidemiology of transmission of cytomegalovirus from mother to preterm infant by breastfeeding. *Lancet* 357:513-518.

11. Revello, M. G., M. Zavattoni, A. Sarasini, et al. 1998. Human cytomegalovirus in blood of immunocompetent persons during primary infection: prognostic implications for pregnancy. *J Infect Dis* 177:1170-1175.

12. Kovacs, A., M. Schluchter, K. Easley, et al. 1999. Cytomegalovirus infection and HIV-1 disease progression in infants born to HIV-1-infected women. Pediatric Pulmonary and Cardiovascular Complications of Vertically Transmitted HIV Infection Study Group. *N Engl J Med* 341:77-84.

13. Doyle, M., J. T. Atkins, and I. R. Rivera-Matos. 1996. Congenital cytomegalovirus infection in infants infected with human immunodeficiency virus type 1. *Pediatr Infect Dis J* 15:1102-1106.

14. Nigro, G., A. Krzysztofiak, G. C. Gattinara, et al. 1996. Rapid progression of HIV disease in children with cytomegalovirus DNAemia. *Aids* 10:1127-1133.

15. Slyker, J. A., B. Lohman-Payne, S. Rowland-Jones, et al. 2008. The detection of cytomegalovirus DNA in maternal plasma is associated with mortalty in HIV-1 infected women and their infants. *submitted*.

16. RochePharmaceuticals. 2008. Cytovene (ganciclovir) product insert.

17. Lowance, D., H. H. Neumayer, C. M. Legendre, et al. 1999. Valacyclovir for the prevention of cytomegalovirus disease after renal transplantation. International Valacyclovir Cytomegalovirus Prophylaxis Transplantation Study Group. *N Engl J Med* 340:1462-1470.

18. Hodson, E. M., J. C. Craig, G. F. Strippoli, et al. 2008. Antiviral medications for preventing cytomegalovirus disease in solid organ transplant recipients. *Cochrane Database Syst Rev*:CD003774.

19. Fiddian, P., C. A. Sabin, and P. D. Griffiths. 2002. Valacyclovir provides optimum acyclovir exposure for prevention of cytomegalovirus and related outcomes after organ transplantation. *J Infect Dis* 186 Suppl 1:S110-115.

20. Emery, V. C., C. Sabin, J. E. Feinberg, et al. 1999. Quantitative effects of valacyclovir on the replication of cytomegalovirus (CMV) in persons with advanced human immunodeficiency virus disease: baseline CMV load dictates time to disease and survival. The AIDS Clinical Trials Group 204/Glaxo Wellcome 123-014 International CMV Prophylaxis Study Group. *J Infect Dis* 180:695-701.

21. Feinberg, J. E., S. Hurwitz, D. Cooper, et al. 1998. A randomized, double-blind trial of valaciclovir prophylaxis for cytomegalovirus disease in patients with advanced human immunodeficiency virus infection. AIDS Clinical Trials Group Protocol 204/Glaxo Wellcome 123-014 International CMV Prophylaxis Study Group. *J Infect Dis* 177:48-56.

22. Reddy, S. P., A. Handa, L. Tan, et al. 2003. Low-dose valaciclovir prophylaxis against cytomegalovirus disease in renal transplant recipients. *Transpl Int* 16:726-729.

23. Kimberlin, D. F., S. Weller, R. J. Whitley, et al. 1998. Pharmacokinetics of oral valacyclovir and acyclovir in late pregnancy. *Am J Obstet Gynecol* 179:846-851.

24. Meyer, L. J., P. de Miranda, N. Sheth, et al. 1988. Acyclovir in human breast milk. *Am J Obstet Gynecol* 158:586-588.

25. Lau, R. J., M. G. Emery, and R. E. Galinsky. 1987. Unexpected accumulation of acyclovir in breast milk with estimation of infant exposure. *Obstet Gynecol* 69:468-471.

26. Ashley, R. L., J. Militoni, F. Lee, et al. 1988. Comparison of Western blot (immunoblot) and glycoprotein G-specific immunodot enzyme assay for detecting antibodies to herpes simplex virus types 1 and 2 in human sera. *J Clin Microbiol* 26:662-667.

27. DeVange Panteleeff, D., S. Emery, B. A. Richardson, et al. 2002. Validation of performance of the gen-probe human immunodeficiency virus type 1 viral load assay with genital swabs and breast milk samples. *J Clin Microbiol* 40:3929-3937.

28. Jerome, K. R., M. L. Huang, A. Wald, et al. 2002. Quantitative stability of DNA after extended storage of clinical specimens as determined by real-time PCR. *J Clin Microbiol* 40:2609-2611.

29. Wald, A., M. L. Huang, D. Carrell, et al. 2003. Polymerase chain reaction for detection of herpes simplex virus (HSV) DNA on mucosal surfaces: comparison with HSV isolation in cell culture. *J Infect Dis* 188:1345-1351.

30. Barbi, M., S. Binda, V. Primache, et al. 2000. Cytomegalovirus DNA detection in Guthrie cards: a powerful tool for diagnosing congenital infection. *J Clin Virol* 17:159-165.

31. Mattes, F. M., E. G. Hainsworth, A. F. Hassan-Walker, et al. 2005. Kinetics of cytomegalovirus load decrease in solid-organ transplant recipients after preemptive therapy with valganciclovir. *J Infect Dis* 191:89-92.

**I. BUDGET**

1. Personnel: no support requested
2. Patient costs: no support requested
3. Supplies and equipment: $23,404
4. Animal acquisition: not applicable
5. Travel & accommodation: $1596
6. Transport: no support requested
7. Operating expenses: no support requested
8. Consultancy: not applicable
9. Miscellaneous: no support requested
10. Contingency no support requested
